# Infrared and Visible Image Fusion via Lightweight Semantic Prior Encoding and Cross-Attention Fusion

**DOI:** 10.3390/s26134300

**Published:** 2026-07-06

**Authors:** Xun Zhang, Di Wu, Jianqi Li, Na Cui

**Affiliations:** 1 College of Intelligent Systems Science and Engineering, Harbin Engineering University, Harbin 150001, China; zhangxun2008@hrbeu.edu.cn (X.Z.); heuwdvision@sina.com (D.W.); 13193885307@hrbeu.edu.cn (N.C.); 2 Qingdao Innovation and Development Center, Harbin Engineering University, Qingdao 266000, China

**Keywords:** image fusion, semantic prior, multi-scale extraction, cross-attention

## Abstract

Infrared (IR) and visible image fusion aims to synthesize a composite representation that integrates the thermal target saliency of IR imagery with the textural richness of visible imagery. Existing deep learning-based methods have achieved promising progress in this field. However, they either operate at the pixel level without semantic priors, or rely on segmentation supervision to obtain such priors. Both approaches limit their practicality and performance in complex scenes. To design a lightweight fusion network that leverages semantic priors without segmentation supervision, we propose SPE2Fusion, a semantic prior-driven fusion network that operates through a dual-stage semantic injection paradigm. Specifically, a lightweight semantic encoder is designed to extract multi-scale scene priors in an end-to-end manner optimized solely by the fusion loss, without requiring segmentation mask annotations. Then, these priors are injected at two complementary stages: the Efficient Semantic Feature Awareness (ESFA) module applies spatially adaptive attention at the encoding stage to amplify semantically salient regions, while the Efficient Semantic Feature Embedding (ESFE) module applies semantically conditioned spatial normalization at the decoding stage to ensure coherent texture reconstruction. Finally, a bidirectional cross-attention fusion block is introduced to integrate complementary cross-modal features under this dual semantic guidance. The network is supervised by a multi-constraint loss combining gradient fidelity, intensity preservation, and structural similarity terms. Comprehensive experiments on the MSRS, LLVIP, and RoadScene benchmarks demonstrate that SPE2Fusion achieves state-of-the-art performance against representative methods (e.g., CrossFuse and DDBFusion), ranking first on four of six metrics on the MSRS test set, specifically EN (6.70), $Q^{AB/F}$ (0.86), AG (6.06), and SD (43.44), while maintaining strong generalization on unseen datasets without domain adaptation.

## 1. Introduction

Infrared (IR) and visible image fusion is a fundamental technique in multimodal sensor information processing. In multi-sensor imaging systems, IR sensors based on microbolometer or photon-counting detector arrays capture thermal radiation in the long-wave infrared (8–14 μm) band, effectively highlighting heat-emitting targets such as pedestrians and vehicles under extreme conditions including low illumination, dense fog, and smoke. However, constrained by detector pitch, thermal sensitivity, and diffraction limits, IR images typically exhibit low spatial resolution and lack fine geometric textures. Visible sensors operating in the 0.4–0.7 μm range capture reflected light with high spatial resolution and rich textural details that align with human visual perception; yet their imaging quality degrades severely under insufficient lighting or strong light interference. By fusing the complementary physical characteristics of these two sensor modalities, a single composite representation can be generated that preserves both the thermal target saliency of the IR modality and the textural fidelity of the visible modality. This fused representation holds considerable potential for enhancing scene understanding in applications such as autonomous driving perception and intelligent video surveillance [1,2].

Early approaches to image fusion, such as those based on multi-scale decomposition, guided filtering [3], and sparse representation, drove initial progress in this field [4]. Nevertheless, these methods heavily rely on manually designed transformation operators and predefined fusion rules, which fundamentally limits their flexibility and adaptability to non-linear cross-modal differences. In recent years, deep learning techniques, particularly Convolutional Neural Networks (CNNs) and Generative Adversarial Networks (GANs), have become mainstream in the fusion domain. Benefiting from their powerful non-linear fitting capabilities, these data-driven models can automatically learn complex mapping relationships between modalities, effectively overcoming the inherent limitations of hand-crafted priors.

Despite these advancements, existing deep fusion models still encounter significant bottlenecks regarding the effective utilization of semantic information and computational efficiency. Representative CNN-based fusion networks such as dense fusion network (DenseFuse [5]) and unified fusion framework (U2Fusion [6]), as well as GAN-based approaches such as fusion network-based GAN (FusionGAN [7]), primarily operate on pixel-level mappings (intensity, structural, or gradient differences) without incorporating high-level semantic priors during the generation process. This absence of semantic awareness can lead to suboptimal target saliency, edge degradation, and semantic inconsistencies, where fused foreground regions exhibit textures mismatched with their object categories or structural boundaries fail to align with semantically meaningful contours, particularly in complex traffic scenarios. More recent methods have sought to bridge this gap: the semantic-aware fusion network (SeAFusion [8]) pioneers the use of semantic loss from a segmentation branch as auxiliary supervision, the correlation-driven decomposition fusion network (CDDFuse [9]) employs correlation-driven dual-branch feature decomposition to separately model common and differential features, and the target-aware dual adversarial learning framework (TarDAL [10]) explores textual semantic priors via cross-modal distillation. While these approaches demonstrably improve semantic coherence, they share a common limitation: they rely on auxiliary modules with substantial parameter overhead, such as large-scale segmentation decoders trained with dense mask annotations. This reliance introduces redundant computational burden and limits their applicability to latency-sensitive deployment scenarios.

To address the aforementioned challenges and design a lightweight fusion network that leverages semantic priors without segmentation supervision, a Semantic Priors Efficient Fusion Network (SPE2Fusion) is proposed. In contrast to methods that rely on explicit segmentation supervision, SPE2Fusion introduces a lightweight semantic encoder that extracts multi-scale scene priors without requiring segmentation mask annotations. The key insight is that cross-modal discrepancies between IR and visible modalities correlate with semantically meaningful scene structures: thermal targets, object boundaries, and foreground regions each exhibit distinctive multi-modal signatures. By optimizing the encoder end-to-end through the fusion loss, the network learns to discover these structurally salient patterns autonomously, effectively producing features that are both spatially localized and semantically relevant without access to object-level category annotations. These learned priors are injected into the network at two complementary stages: an Efficient Semantic Feature Awareness (ESFA) module at the encoding stage applies spatially adaptive attention to amplify semantically salient features while suppressing background clutter, and an Efficient Semantic Feature Embedding (ESFE) module at the decoding stage modulates the upsampling process via spatially adaptive normalization conditioned on the semantic prior, ensuring that reconstructed textures remain semantically coherent. The entire network is trained end-to-end with a multi-constraint loss function that combines gradient fidelity, intensity fidelity, and structural similarity terms to jointly optimize edge sharpness, luminance integrity, and perceptual quality. This dual-stage injection paradigm is designed to enhance target saliency and preserve structural detail while maintaining a favorable balance between fusion quality and computational cost.

The main contributions of this paper are summarized as follows:

- A semantic prior-driven image fusion network, SPE2Fusion, is proposed to narrow the gap between low-level pixel reconstruction and high-level semantic representation. The network employs a lightweight semantic encoder that learns to extract multi-scale priors relevant to scene structure through end-to-end fusion loss optimization, without requiring segmentation mask supervision.
- A dual-stage injection paradigm is designed, comprising the ESFA module at the encoding stage for spatially adaptive feature enhancement and the ESFE module at the decoding stage for semantically conditioned image reconstruction. This paradigm provides a lightweight, modular approach for embedding learned priors into encoder-decoder fusion architectures, applicable beyond the specific network presented here.
- A multi-constraint loss function is formulated that combines gradient fidelity, intensity fidelity, and structural similarity terms to jointly optimize edge sharpness, luminance consistency, and perceptual quality, providing balanced supervision across complementary quality dimensions.

The remainder of this paper is organized as follows. Section 2 reviews related work on CNN-, GAN-, and Transformer-based infrared and visible image fusion methods. Section 3 describes the proposed SPE2Fusion architecture in detail, including the semantic encoder, the ESFA and ESFE modules, the cross-modal fusion strategy, and the training objective. Section 4 presents the experimental setup and a systematic evaluation covering qualitative comparisons, quantitative benchmarks on multiple datasets, generalization analysis, and ablation studies. Section 5 discusses the experimental findings, component interactions, and limitations of the proposed approach, and concludes with a summary of contributions and directions for future research.

## 2. Related Work

### 2.1. CNN- and GAN-Based Fusion Methods

Early deep learning approaches to infrared and visible image fusion relied primarily on autoencoder and CNN architectures. Li et al. proposed DenseFuse [5], which employs a dense block within an autoencoder to aggregate multi-layer features and applies manually designed fusion rules to combine the encoded representations. Li et al. developed a residual nest fusion network (RFN-Nest [11]), an end-to-end residual fusion network that eliminates handcrafted fusion rules by jointly learning a fusion strategy within a nested architecture. Zhang et al. proposed a general-purpose image fusion framework (IFCNN [12]) based on convolutional operations that directly fuses source images in the spatial domain. Liu et al. [13] demonstrated the effectiveness of multi-scale supervision and implicit guidance for robust feature learning in complex scenes. Xu et al. introduced U2Fusion [6], a unified unsupervised fusion model that adaptively preserves source information guided by an information measurement criterion. However, its reliance on hand-crafted information measures limits its adaptability to complex scenarios, resulting in the loss of modality-specific details. Zhang et al. [14] introduced dual decomposition and Bézier curves for infared and visible image fusion. Despite their effectiveness, these CNN-based methods are constrained by local receptive fields, limiting their ability to capture long-range contextual dependencies across modalities.

Generative adversarial networks (GANs) have also been widely explored for image fusion. Ma et al. proposed FusionGAN [7], which formulates fusion as an adversarial process in which a generator produces fused images that simultaneously preserve infrared thermal saliency and visible textural detail. Ma et al. [15] introduced multiclassification constraints-based GAN (GANMcC) to transform the fusion problem into a simultaneous multi-distribution estimation task, improving modality balance. Tang et al. proposed SeAFusion [8], which pioneers the use of a semantic segmentation branch as auxiliary supervision to guide the fusion process, representing an early effort to incorporate high-level semantic priors. The same group further proposed a novel illumination-aware loss to progressively adapt the fusion strategy (PIAFusion [16]) across diverse lighting conditions. Zhao et al. proposed CDDFuse [9], a correlation-driven dual-branch decomposition network that separately models modality-shared and modality-specific features. Liu et al. proposed TarDAL [10], which leverages cross-modal distillation from a detection network to align fused features with downstream task semantics. While these approaches improve fusion quality, they commonly rely on auxiliary supervision modules with substantial parameter overhead, limiting applicability in latency-sensitive scenarios.

### 2.2. Transformer-Based Fusion Methods

The strong global modeling capability of the Transformer architecture has motivated its adoption in image fusion. Wang et al. [17], proposed a residual Swin Transformer network that leverages shifted-window self-attention to model long-range contextual dependencies while preserving local detail. Ma et al. [18] proposed a cross-domain long-range learning framework comprising domain-internal and cross-domain fusion units based on self-attention and cross-attention, effectively addressing limitations in global context modeling. Tang et al. [19] proposed an end-to-end dual-attention Transformer fusion model that simultaneously captures local and global contextual information, retaining important local features while integrating global structure. Wang et al. [20] proposed a method that alternately learns local and global features through adaptive interactive Transformer learning, addressing limitations in cross-modal interaction and local–global relation modeling. Tang et al. [21] proposed an interactive Transformer framework that dynamically mines complementary information across modalities and builds long-range dependencies through cross-modal attention blocks. Cai et al. [22] proposed a model that introduces intra-modal and cross-modal feature mixing modules using residual MLP and depthwise separable convolution to integrate complementary information between modalities. Zhao et al. proposed a fusion network combining a residual interactive Transformer with a cross-attention fusion module, achieving effective integration of global and local features across infrared and visible modalities [23]. Beyond attention-based architectures, Li et al. [24] proposed a method that decouples features into modality-shared and modality-specific components via representation learning to improve cross-modal complementarity. Wang et al. [25] explored mask-guided cross-modal fusion in visible-infrared tasks with a state space model. Li et al. [26] designed a novel cross-attention fusion (CrossFuse) network-based transformer for infrared and visible image fusion. More recently, Zhao et al. [27] formulated fusion as an iterative denoising diffusion process conditioned on both source modalities, and equivariant methods [28] have been explored to enforce geometric consistency across modalities as a structural inductive bias.

Although Transformer-based methods demonstrate significant improvements in global context modeling and cross-modal feature integration, they share common limitations: substantial model sizes, high computational cost, and the absence of explicit high-level semantic guidance during feature extraction and reconstruction. Moreover, their internal decision-making process remains largely opaque, which hinders interpretability and makes it difficult to diagnose failure cases in complex scenes. These shortcomings motivate the design of SPE2Fusion, which introduces a lightweight semantic encoder to implicitly extract multi-scale scene priors end-to-end, enabling semantically guided fusion without the overhead of explicit segmentation supervision or large auxiliary modules. Notably, unlike these methods (e.g., SeAFusion and TarDal) that depend on external annotations or pre-trained models, our approach learns semantic priors solely through fusion loss optimization, offering a more flexible and annotation-efficient paradigm.

## 3. Proposed Method

### 3.1. Overall Architecture

**Figure 1:**
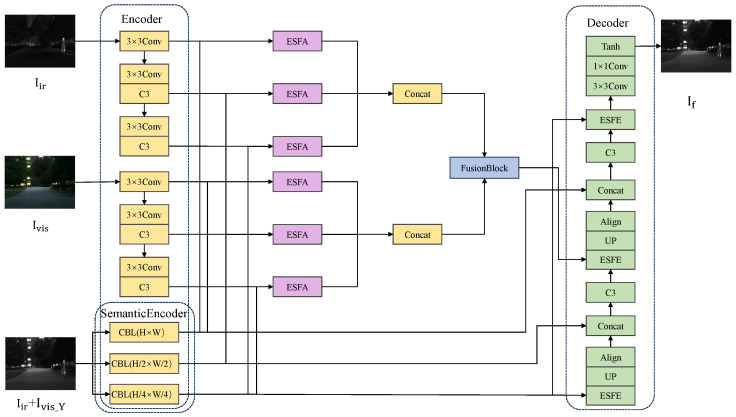
Overall architecture of the proposed SPE2Fusion. The IR and visible encoders extract multi-scale features, which are fused by the bidirectional cross-attention fusion block under the guidance of ESFA modules. The semantic encoder simultaneously extracts multi-scale priors, which guide ESFA at the encoding stage and further feed into ESFE for semantically conditioned decoding and final reconstruction.

The proposed SPE2Fusion adopts an encoder–fusion–decoder framework augmented with a dedicated semantic guidance pathway. As illustrated in Figure 1, the architecture comprises five functional modules: (1) modal encoders that independently extract multi-scale features from the visible and infrared images; (2) a lightweight semantic encoder that produces multi-scale scene priors to guide the fusion process; (3) Efficient Semantic Feature Awareness (ESFA) modules that inject semantic guidance into encoder features via spatially adaptive attention; (4) cross-modal fusion modules (FusionBlock) that fuse semantically enhanced features at each scale through a cross-attention mechanism; and (5) a semantic decoder that reconstructs the fused image with Efficient Semantic Feature Embedding (ESFE) modules providing semantic-aware modulation during upsampling.

The visible and infrared encoders share an identical structure but maintain independent parameters to accommodate the distinct characteristics of the two modalities. Each encoder consists of three sequential stages. The first stage applies a 3×3 convolution (stride 1) followed by a CSP bottleneck block (C3 module) [29], producing 32-channel features at the original resolution. The second stage uses a 3×3 convolution with stride 2 for downsampling, followed by a C3 module, yielding 64-channel features at 1/2 resolution. The third stage further downsamples via another strided 3×3 convolution and C3 module, outputting 128-channel features at 1/4 resolution. This design yields three spatial scales (full, 1/2, and 1/4 of the original resolution, with channel dimensions of 32, 64, and 128), capturing multi-scale local textures and structural information. The infrared branch is inherently biased toward thermal target responses, while the visible branch focuses on textural details and spatial structures, providing complementary feature representations for subsequent fusion.

The core innovation of SPE2Fusion lies in the semantic guidance pathway, which operates across the full feature-extraction-to-image-reconstruction pipeline. The data flow proceeds as follows. First, the visible and infrared encoders independently extract multi-scale features from their respective inputs. At each scale, ESFA modules inject the corresponding semantic prior into the encoder features, producing semantically enhanced representations. These are then fused across modalities by the cross-modal FusionBlock. Finally, the semantic decoder progressively upsamples the fused features back to the original resolution, with ESFE modules at each upsampling stage ensuring that the reconstructed image remains semantically coherent. This dual-stage injection paradigm (semantic modulation before fusion and semantic guidance during reconstruction) enables the network to maintain effective semantic awareness without relying on segmentation mask supervision.

At each scale, the semantically enhanced visible and infrared features are fused through a Transformer-based FusionBlock. The module employs bidirectional cross-attention with 4-head scaled dot-product attention [30]: visible features serve as queries attending to infrared key-value pairs, capturing thermal target cues, while infrared features serve as queries attending to visible key-value pairs, incorporating textural details. Each cross-attention path follows the standard Transformer block structure: layer normalization precedes the multi-head attention computation; the attended output is combined with the branch input via a learnable-weighted residual connection (parameterized by learnable scalars). A subsequent feed-forward MLP sub-layer, also preceded by layer normalization and followed by a second learnable-weighted residual connection, further refines the attended representation. The outputs of the two cross-attention branches are element-wise summed to yield the fused feature at that scale. This fusion operation is performed independently at each of the three scales, and the resulting multi-scale fused features are passed to the semantic decoder for reconstruction.

### 3.2. Lightweight Semantic Encoder

To provide unified semantic guidance with minimal computational overhead, SPE2Fusion employs a lightweight semantic encoder that extracts multi-scale scene priors without relying on segmentation mask supervision. The semantic encoder is trained end-to-end as part of the full network, with its parameters optimized solely by the gradient signals propagated from the fusion loss function (described in Section 3.5). A key question underlying this design is whether features learned without explicit semantic labels can carry semantically meaningful information. The underlying mechanism relies on the observation that cross-modal discrepancies between IR and visible modalities are not uniform across a scene: thermal targets (e.g., pedestrians, vehicles) exhibit strong IR responses but low visible texture, while background regions (e.g., sky, vegetation) show the opposite pattern. By optimizing for fusion quality, which requires identifying which regions to emphasize from which modality, the encoder is driven to discover these structurally salient patterns. The resulting features, while not corresponding to explicit category labels, correlate strongly with scene structures that are semantically relevant for the fusion task. This implicit learning mechanism enables the encoder to produce features that capture functionally meaningful scene structure without requiring explicit segmentation labels.

The input to the semantic encoder is the channel-wise concatenation of the single-channel infrared image and the Y channel (luminance) of the visible image. The use of the Y channel rather than the full RGB representation is motivated by two considerations. First, infrared images inherently capture thermal radiation intensity, which correlates naturally with luminance rather than chrominance. Second, operating on a single luminance channel significantly reduces input dimensionality and computational cost.

**Figure 2:**
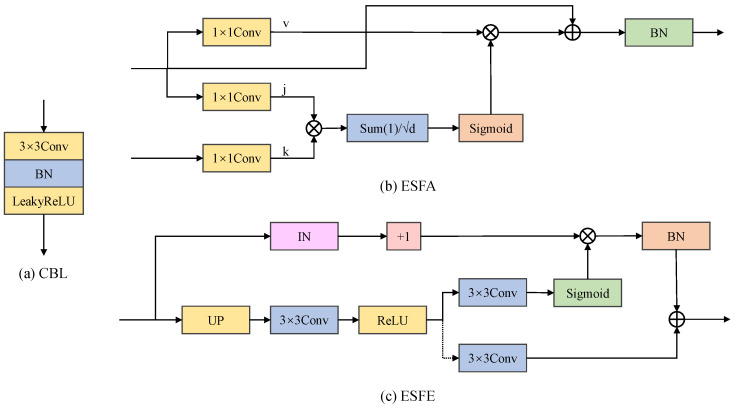
Architectures of the three proposed modules. (**a**) CBL block: the basic convolutional building block (3×3 Conv + BN + LeakyReLU) used in the semantic encoder. (**b**) ESFA module: semantic-prior-guided spatial attention applied at the encoding stage. (**c**) ESFE module: semantically conditioned feature modulation applied at each decoder scale.

As shown in Figure 2a, the semantic encoder adopts a fully convolutional architecture comprising three sequential CBL stages. Each CBL block consists of a 3×3 convolution, batch normalization, and LeakyReLU activation applied in sequence. The strides of the three stages are set to s={1,2,2}, yielding a three-scale feature pyramid whose spatial resolutions match those of the modal encoders:

(1) $$S_{1},S_{2},S_{3}=\mathrm{SemanticEncoder}\left(\left[I_{ir},Y_{vis}\right]\right)$$

where $S_{1}\in R^{B\times C_{s}\times H\times W}$, $S_{2}\in R^{B\times 2C_{s}\times H/2\times W/2}$, and $S_{3}\in R^{B\times 4C_{s}\times H/4\times W/4}$. They are multi-scale semantic priors extracted by the semantic encoder. These semantic priors guide the ESFA module to fuse IR and visible features at each corresponding scale, and are simultaneously fed into the ESFE module to support multi-scale feature decoding for final image reconstruction. The base channel count $C_{s}$ is set to 16, approximately half that of the modal encoders (C=32). This asymmetric channel design, inspired by the spatial path of BiSeNet [31], preserves sufficient spatial guidance capacity while substantially reducing parameter overhead.

The three semantic scales serve complementary roles: $S_{1}$ retains high spatial resolution and rich local detail, suitable for fine-grained semantic enhancement of shallow encoder features; $S_{2}$ and $S_{3}$, with progressively larger receptive fields through downsampling, encode higher-level semantic context, making them more effective for global semantic modulation of deeper features. This multi-scale design structurally aligns with the decoder’s progressive upsampling process, ensuring continuous semantic guidance throughout image reconstruction. With only 16 base channels and no fully connected layers, the semantic encoder introduces only 0.67 M additional parameters (approximately 38% of the 1.76 M total model size, compared to 1.09 M for the baseline encoder–fusion–decoder without the semantic pathway).

### 3.3. Efficient Semantic Feature Awareness Module

Conventional encoder architectures extract features without regard to semantic content, treating foreground targets and background regions with uniform operations. This lack of semantic discrimination causes the subsequent fusion module to aggregate information indiscriminately over semantically irrelevant regions, potentially diluting target saliency and amplifying background noise. The ESFA module addresses this limitation by injecting semantic priors into the encoder feature stream before fusion, enabling the network to preferentially enhance semantically salient regions.

As shown in Figure 2b, at each encoder scale, ESFA receives the semantic prior $S_{n}$ and the image feature $F_{n}$ (from either the visible or infrared encoder at the corresponding scale). Three learnable 1×1 convolution mappings are applied to construct the semantic key *k*, image key *j*, and value feature *v*:

(2) $$k={\mathrm{Conv}}_{k}\left(S_{n}\right)\in R^{B\times d\times H^{\prime }\times W^{\prime }}$$

(3) $$j={\mathrm{Conv}}_{j}\left(F_{n}\right)\in R^{B\times d\times H^{\prime }\times W^{\prime }}$$

(4) $$v={\mathrm{Conv}}_{v}\left(F_{n}\right)\in R^{B\times C\times H^{\prime }\times W^{\prime }}$$

where *d* denotes the feature projection dimension, set equal to the image feature channel count at each scale (i.e., d=C at scale 1, d=2C at scale 2, and d=4C at scale 3), and $H^{\prime },W^{\prime }$ the spatial dimensions at the current scale. A spatial attention map is computed through element-wise matching of the semantic and image keys at each spatial position, followed by channel-wise aggregation and sigmoid activation:

(5) $$A_{n}=\sigma \;\left(\frac{\sum_{c=1}^{d}\left(k⊙j\right)}{\sqrt{d}}\right)\in R^{B\times 1\times H^{\prime }\times W^{\prime }}$$

where σ denotes the sigmoid function and ⊙ element-wise multiplication. The scaling factor $\sqrt{d}$ stabilizes training by preventing dot-product magnitudes from growing excessively large. Without this factor, the input to the sigmoid function would be pushed into its saturated regions, leading to extremely small gradients that hinder effective feature learning and slow down convergence.

The attention map is then applied to the value feature via element-wise weighting with a residual connection:

(6) $${\tilde{F}}_{n}=\mathrm{BN}\left(v⊙A_{n}+F_{n}\right)$$

where BN denotes batch normalization. The residual connection ensures that semantic priors are injected as a complementary modulation rather than a replacement, preserving the original feature structure while selectively amplifying semantically relevant responses.

This element-wise spatial attention design deliberately avoids constructing the full HW×HW spatial correlation matrix required by standard self-attention, reducing spatial complexity from $O(H^{2}W^{2})$ to O(HW). Rather than explicitly modeling long-range spatial dependencies, ESFA leverages the semantic prior as a learned spatial saliency map that performs position-level modulation of image features, achieving an effective trade-off between semantic enhancement capability and computational efficiency for high-resolution fusion tasks.

### 3.4. Efficient Semantic Feature Embedding Module

While ESFA operates at the encoding stage to filter and enhance features before fusion, the ESFE module complements it at the decoding stage by making the image reconstruction process semantically aware. During progressive upsampling, a conventional decoder lacking semantic guidance tends to apply a uniform reconstruction strategy across all spatial regions, which can lead to blurred target boundaries and inconsistent foreground-background quality. ESFE addresses this by conditioning the upsampling process on the semantic prior through spatially adaptive normalization.

As illustrated in Figure 2c, at each decoding scale, ESFE receives the corresponding semantic prior $S_{n}$ and the fused feature $F_{n}^{\mathrm{fuse}}$ from the cross-modal fusion module. When the spatial dimensions of $S_{n}$ and $F_{n}^{\mathrm{fuse}}$ differ, $S_{n}$ is first aligned to the target resolution via bilinear interpolation. The aligned semantic prior is then encoded via a shared 3×3 convolution mapping with ReLU activation to produce a compact semantic embedding *E*, whose channel dimension $C_{\mathrm{mid}}$ is set equal to the fused feature channel count $C_{n}$ at the current scale:

(7) $$E=\mathrm{ReLU}\;\left(\mathrm{Conv}(S_{n})\right)\in R^{B\times C_{\mathrm{mid}}\times H^{\prime }\times W^{\prime }}$$

Two parallel convolution branches then generate spatially varying modulation parameters from this embedding:

(8) $$\gamma =\sigma \;\left({\mathrm{Conv}}_{\gamma }\left(E\right)\right)\in R^{B\times C_{n}\times H^{\prime }\times W^{\prime }}$$

(9) $$\beta ={\mathrm{Conv}}_{\beta }\left(E\right)\in R^{B\times C_{n}\times H^{\prime }\times W^{\prime }}$$

where γ is a multiplicative modulation factor constrained to (0,1) via sigmoid activation, controlling the intensity of feature responses at each spatial position, and β is an additive bias term providing compensatory offset for fine-grained structure refinement.

The fused feature is then modulated through a spatially adaptive normalization operation inspired by SPADE [32]:

(10) $${\hat{F}}_{n}^{\mathrm{fuse}}=\mathrm{BN}\;\left(\left(\mathrm{IN}(F_{n}^{\mathrm{fuse}})+1\right)⊙\gamma +\beta \right)$$

where IN denotes instance normalization and BN batch normalization. Instance normalization first eliminates inter-channel statistical discrepancies, bringing features from different modalities to a unified scale and mitigating distribution shift. The additive constant +1 after instance normalization shifts the feature mean to approximately 1, ensuring that when the multiplicative factor γ∈(0,1) approaches zero, the base feature representation is preserved rather than entirely suppressed, thereby stabilizing the modulation behavior. The semantic modulation parameters then perform position-specific adjustment: γ selectively amplifies target-region responses and suppresses background clutter according to the semantic prior, while β refines local structural details to preserve textural fidelity. This dual-parameter design enables semantic information to be embedded into the decoding process in a continuous and stable manner without disrupting the spatial structure of the original fused features.

The synergistic interaction between ESFA and ESFE establishes a coherent semantic guidance pipeline: ESFA ensures that features entering the fusion module are biased toward semantically meaningful regions, while ESFE ensures that the reconstructed image maintains this semantic coherence at the pixel level. Together, they resolve the semantic blindness that characterizes conventional encoder–fusion–decoder networks.

### 3.5. Multi-Constraint Loss Function

Since no ground-truth fused image exists for infrared–visible fusion, SPE2Fusion is trained with an unsupervised multi-constraint loss function that jointly optimizes the fused output from three complementary quality dimensions: gradient fidelity, intensity fidelity, and structural similarity.

**Gradient loss.** To preserve edge sharpness and prevent the over-smoothing commonly introduced by feature averaging in fusion, the gradient loss constrains the fused image to maintain gradient magnitudes comparable to the strongest responses in either source image. The gradient magnitude |∇I| is computed using the Sobel operator, with the horizontal and vertical convolution kernels defined as:

(11) $$G_{x}=\left[\begin{array}{ccc} -1 & 0 & 1\\ -2 & 0 & 2\\ -1 & 0 & 1 \end{array}\right],\;G_{y}=\left[\begin{array}{ccc} 1 & 2 & 1\\ 0 & 0 & 0\\ -1 & -2 & -1 \end{array}\right]$$

The gradient magnitude is then approximated as $\left|\nabla I\right|=|I*G_{x}|+|I*G_{y}|$, where ∗ denotes the convolution operation. The gradient loss takes the following form:

(12) $$L_{\mathrm{grad}}={∥\max(|\nabla Y_{\mathrm{vis}}|,|\nabla I_{\mathrm{ir}}|)-|\nabla I_{\mathrm{fuse}}|∥}_{1}$$

The element-wise maximum operation selects the stronger gradient response from the two source modalities at each pixel. By constraining the fused gradient to match this upper bound rather than the average, the loss compels the network to preserve the sharpest available edge information at every spatial position, effectively preventing edge degradation. The $\ell_{1}$ norm is employed for its robustness to gradient outliers and its tendency to produce sparser error distributions, which benefits fine-grained texture retention.

**Intensity loss.** To maintain luminance integrity and thermal target saliency, the intensity loss constrains the fused image’s pixel intensity distribution:

(13) $$L_{\mathrm{int}}={∥\max\left(Y_{\mathrm{vis}},I_{\mathrm{ir}}\right)-I_{\mathrm{fuse}}∥}_{1}$$

where $Y_{\mathrm{vis}}$ denotes the Y channel of the visible image after conversion from RGB to YCbCr color space, chosen because the Y channel’s definition aligns with human luminance perception. The element-wise maximum reference plays a physically meaningful role: infrared images exhibit high intensity in thermal target regions, while visible images provide higher luminance in well-lit background areas. The maximum operation ensures that neither modality’s brightness information is compressed—thermal targets retain their high responses, and background regions preserve natural luminance levels.

**Structural similarity loss.** Complementing the pixel-level gradient and intensity constraints, the structural similarity loss enforces perceptual-level consistency:

(14) $$L_{\mathrm{ssim}}=1-\mathrm{SSIM}\;\left(I_{\mathrm{fuse}},\max\left(Y_{\mathrm{vis}},I_{\mathrm{ir}}\right)\right)$$

The SSIM index [33] is computed via Gaussian-weighted local statistics that jointly assess luminance, contrast, and structural similarity:

(15) $$\mathrm{SSIM}\left(x,y\right)=\frac{\left(2\mu_{x}\mu_{y}+C_{1}\right)\left(2\sigma_{xy}+C_{2}\right)}{\left(\mu_{x}^{2}+\mu_{y}^{2}+C_{1}\right)\left(\sigma_{x}^{2}+\sigma_{y}^{2}+C_{2}\right)}$$

where $\mu_{x}$, $\mu_{y}$, $\sigma_{x}$, $\sigma_{y}$, and $\sigma_{xy}$ denote local means, standard deviations, and cross-covariance computed through a Gaussian window, with $C_{1}={\left(0.01\right)}^{2}$ and $C_{2}={\left(0.03\right)}^{2}$ as numerical stability constants. This loss effectively suppresses block artifacts and local contrast degradation that may escape pixel-level constraints.

**Total loss.** The overall training objective is a weighted linear combination of the three terms:

(16) $$L_{\mathrm{total}}=\lambda_{\mathrm{grad}}L_{\mathrm{grad}}+\lambda_{\mathrm{int}}L_{\mathrm{int}}+\lambda_{\mathrm{ssim}}L_{\mathrm{ssim}}$$

The weights are selected on the MSRS training set using the average of EN, QAB/F, and AG metrics as the selection criterion, yielding $\lambda_{\mathrm{grad}}=10$, $\lambda_{\mathrm{int}}=1$, and $\lambda_{\mathrm{ssim}}=2$. Gradient fidelity, being most critical for edge sharpness and visual quality, receives the highest weight. The structural similarity term, operating at a perceptual level to enforce overall structural consistency, is assigned a moderate weight. Intensity fidelity, while important for luminance integrity, exerts a relatively indirect influence on perceived quality and is given the base weight. This differentiated weighting scheme ensures that each constraint contributes in proportion to its impact on the fusion task, enabling balanced optimization across edge sharpness, luminance consistency, and perceptual quality.

## 4. Experiments and Results

### 4.1. Experimental Setup

#### 4.1.1. Datasets and Implementation Details

Comprehensive experiments were conducted across multiple public infrared and visible image fusion benchmarks. SPE2Fusion was trained and evaluated on the MSRS dataset [8]. Cross-dataset generalization was assessed without any fine-tuning on 50 randomly selected image pairs from the LLVIP dataset [34] and the RoadScene dataset [35]. MSRS provides paired infrared–visible images with dense pixel-level semantic labels covering both day and night scenes. LLVIP offers high-resolution low-illumination night images featuring pedestrians, vehicles, and traffic elements. RoadScene supplies night-time traffic signal and exposure scenes. These three datasets are complementary in terms of illumination conditions, resolution, and annotation granularity. We adopt all of them to ensure a comprehensive and balanced evaluation across diverse scenarios.

All experiments were performed on a workstation equipped with an NVIDIA RTX 3090 GPU (NVIDIA Corporation, Santa Clara, CA, USA; 24 GB VRAM) and 90 GB of system memory, running Ubuntu 22.04. The implementation used PyTorch 2.3.0 with CUDA 12.1 under Python 3.12. The network was optimized with the Adam optimizer at an initial learning rate of $1\times 10^{-4}$ under an exponential decay schedule, trained for 100 epochs with a batch size of 16.

#### 4.1.2. Comparative Methods

SPE2Fusion is compared against nine representative baselines spanning traditional, CNN-based, and adversarial design paradigms: RFN-Nest [11], SDNet [36], IFCNN [12], U2Fusion [6], FusionGAN [7], GANMcC [15], SeAFusion [8], CrossFuse [26], and DDBFusion [14].

#### 4.1.3. Evaluation Metrics

Six no-reference metrics are adopted for quantitative evaluation, all of which are higher-is-better. Information Entropy (EN) measures the richness of information in the fused image. Average Gradient (AG) quantifies edge sharpness and fine-detail clarity. Spatial Frequency (SF) reflects the overall level of textural activity and detail. Standard Deviation (SD) captures the contrast range of the fused image. Visual Information Fidelity (VIF) [37] measures the degree of source-information preservation relative to the input images. Edge-based Similarity Measure ($Q^{AB/F}$) [38] quantifies the efficiency of gradient information transfer from both source modalities to the fused result.

### 4.2. Qualitative Evaluation

**Figure 3:**
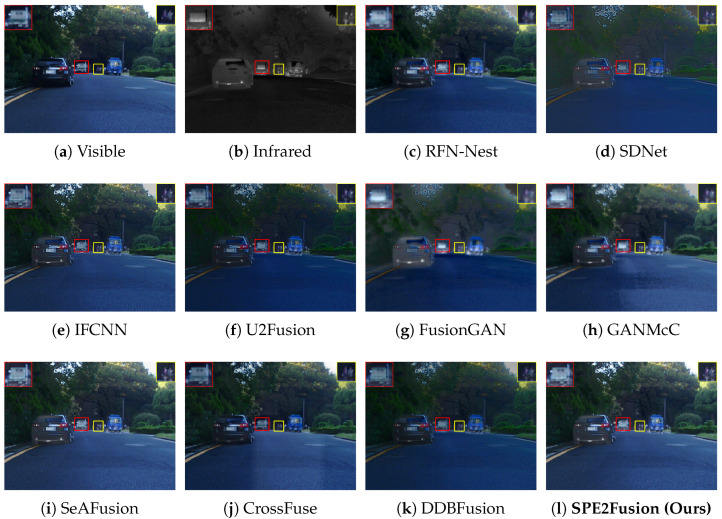
Qualitative comparison on the MSRS daytime scene (00204D). The red box highlights vehicle contours, while the yellow box highlights small targets.

**Figure 4:**
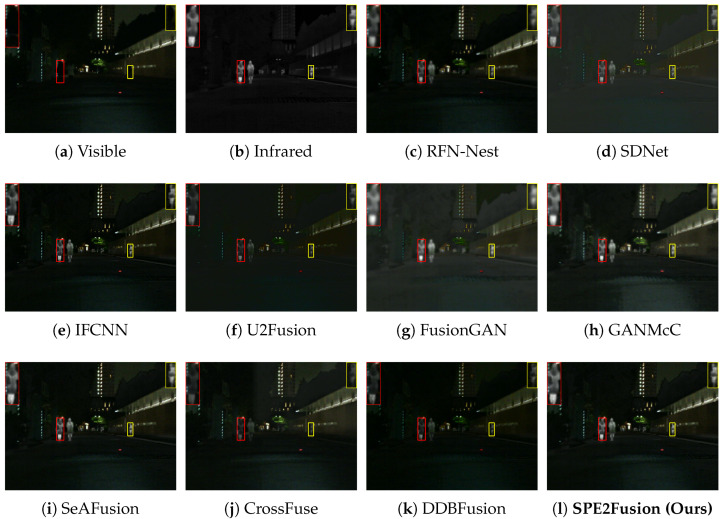
Qualitative comparison on the MSRS nighttime scene (00774N). The red and yellow boxes highlight pedestrians in the dark scene.

Two representative scenes from the MSRS test set are selected for visual comparison: a daytime driving scene (00204D, Figure 3) and a low-light nighttime scene (00774N, Figure 4). Red and yellow bounding boxes annotate regions of interest and are magnified for close-up inspection; red boxes mark semantically salient targets and yellow boxes mark background-texture regions.

In the daytime scene, the visible branch provides rich texture and structural information while the infrared branch highlights thermal targets against the background. Achieving an effective balance between detail retention and target enhancement is therefore the primary challenge. DenseFuse and GTF achieve basic multi-modal integration but produce low-contrast results with blurred distant targets. RFN-Nest and IFCNN preserve structural boundaries reasonably well but provide insufficient infrared-target reinforcement, resulting in reduced target saliency. SDNet introduces brightness non-uniformity and ghosting artifacts that degrade visual naturalness. U2Fusion yields a more balanced overall appearance but remains weak on small, distant targets. FusionGAN and GANMcC visibly enhance target regions at the cost of sacrificing local texture detail, producing local blurring or overall dimming. SeAFusion achieves a reasonable compromise between target enhancement and structural preservation, though local fine-grained details remain insufficiently sharp. SPE2Fusion attains a superior balance: key thermal targets such as vehicles are clearly highlighted, while fine structural details in distant and textured regions are faithfully retained, yielding more natural and informationally complete fusion results.

In the nighttime scene, low visible-image brightness and severe textural degradation place heavy reliance on the infrared branch for target detection. DenseFuse and GTF provide limited overall brightness improvement, with fused results remaining dim. RFN-Nest and IFCNN retain some structural fidelity but under-emphasize infrared targets, leaving target regions insufficiently prominent. SDNet exhibits noticeable brightness non-uniformity and color shift. U2Fusion provides a modest global brightness improvement but remains weak on distant targets. FusionGAN and GANMcC strongly amplify target regions at the expense of introducing noise and losing background structural information. SeAFusion highlights targets effectively but tends toward local over-enhancement or texture blurring. SPE2Fusion substantially improves the visibility of pedestrians and key targets while recovering background structural information, and avoids the over-enhancement and noise artifacts common to competing methods, achieving a well-balanced result across brightness, contrast, and texture dimensions.

### 4.3. Quantitative Comparison on MSRS

Table 1 reports quantitative results on the MSRS test set. Values highlighted in red and blue indicate the best and second-best performance for each metric, respectively.

SPE2Fusion achieves the best performance on four of the six metrics, namely EN (6.70), $Q^{AB/F}$ (0.86), AG (6.06), and SD (43.44), by substantial margins. The $Q^{AB/F}$ advantage is especially pronounced (0.86 vs. 0.66 for the nearest competitor, SeAFusion), indicating a significantly higher rate of gradient information transfer from both source modalities. The leading AG value (6.06 vs. 3.91 for IFCNN) reflects the effectiveness of the semantic-guided fusion strategy in recovering sharp edges at target-region boundaries. SPE2Fusion ranks second on SF (11.43), trailing IFCNN (12.02), and third on VIF (0.89), behind SeAFusion (0.96) and GTF (0.93). These gaps reflect an inherent design trade-off: by directing representational capacity toward semantic-region discrimination and cross-modal feature alignment, the ESFA and ESFE modules prioritize structural coherence over pixel-level source reconstruction fidelity, which SF and VIF predominantly measure. Nevertheless, the model maintains a high level of source-information fidelity while achieving significant advantages across all gradient- and texture-based metrics, demonstrating a well-calibrated balance between perceptual quality and structural preservation. Notably, SeAFusion achieves higher VIF because it incorporates a semantic segmentation network trained with ground-truth labels, which provides stronger supervision for preserving source fidelity and structural naturalness. In contrast, our method extracts semantic priors solely through fusion loss optimization without segmentation annotations, which limits the perceptual fidelity under certain conditions.

Cross-method analysis further highlights the limitations of individual baselines. GTF attains the highest VIF but underperforms substantially on structural and gradient metrics. IFCNN scores well on AG and SF but yields imbalanced overall performance. SeAFusion, the strongest single competitor, falls markedly behind on $Q^{AB/F}$ and AG, confirming that the semantic guidance pathway delivers measurable improvements in target-region detail recovery and cross-modal structural preservation.

### 4.4. Generalization Performance

To probe out-of-distribution robustness, SPE2Fusion trained exclusively on MSRS was evaluated on LLVIP and RoadScene without fine-tuning. These datasets cover scene statistics, lighting conditions, and camera configurations entirely different from the training set, making them stringent tests of generalization.

#### 4.4.1. LLVIP Dataset

**Figure 5:**
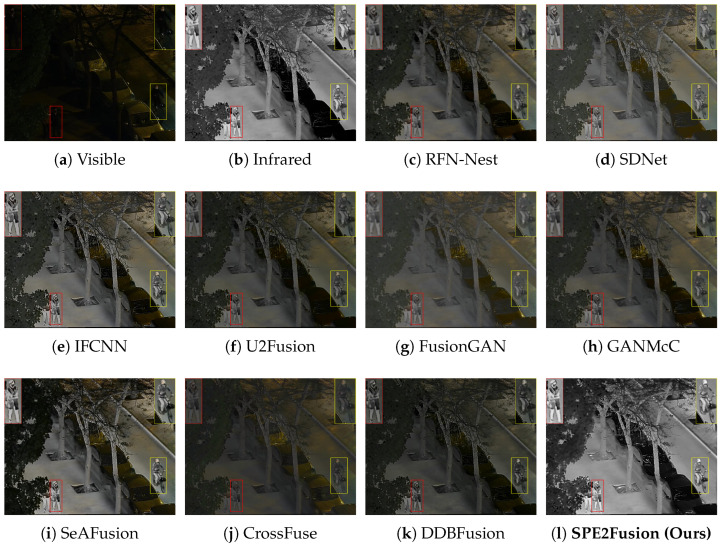
Qualitative comparison on LLVIP image 240015 (low-light nighttime pedestrian scene). The red box highlights a pedestrian, while the yellow box highlights a bicycle edge.

On the LLVIP dataset (Table 2), which presents extreme low-light pedestrian surveillance conditions, SPE2Fusion achieves the best scores on EN (7.46), $Q^{AB/F}$ (0.84), AG (6.22), and VIF (0.79). It also achieves the second-best SD (51.11), trailing only IFCNN (53.59). Although SF (14.33) falls below the peak values of IFCNN (16.23) and SeAFusion (15.12), the model maintains high performance across all remaining metrics. Notably, the lower SF of our method is mainly due to its extreme low-light condition from LLVIP, where the fusion result relies heavily on the infrared modality with limited texture. Qualitatively (Figure 5), SPE2Fusion more clearly delineates pedestrian target contours in challenging low-light conditions while retaining background structural detail, avoiding the over-enhancement or information loss observed in competing methods.

#### 4.4.2. RoadScene Dataset

**Figure 6:**
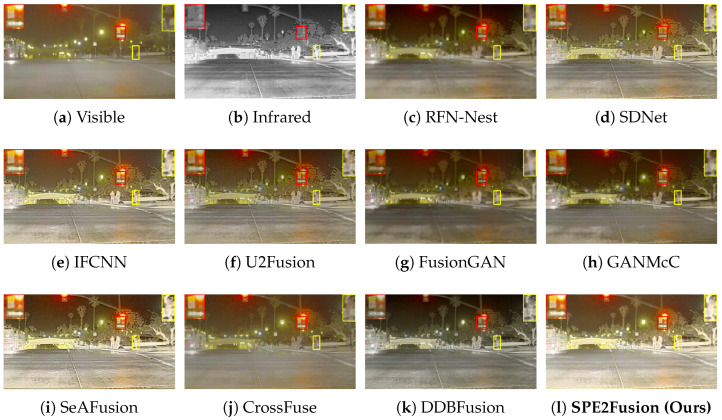
Qualitative comparison on RoadScene image FLIR_09465 (nighttime traffic scene). The red box highlights the traffic light edge, while the yellow box highlights a pedestrian in the dark scene.

On the RoadScene dataset (Table 3), which includes complex nighttime traffic scenarios with high-brightness highlights, SPE2Fusion achieves the best performance on $Q^{AB/F}$ (0.77), SD (53.99), and VIF (0.62), and ranks second on EN (7.44). Although AG (5.48) and SF (9.39) do not reach the best values achieved by SeAFusion (AG: 7.70; SF: 20.21), those peak values are obtained through aggressive local high-frequency enhancement that compromises other quality dimensions. The weaker AG on RoadScene is attributed to strong illumination variations and over-exposed regions, where the gradient distribution deviates from the training data. SPE2Fusion’s leading $Q^{AB/F}$ and SD results confirm its structural preservation advantage, and its strong VIF confirms high source-information fidelity. Qualitatively (Figure 6), SPE2Fusion accurately highlights high-brightness traffic signals while preserving road texture and background structure, avoiding the over-exposure and halo artifacts present in competing methods.

Across both generalization datasets, SPE2Fusion consistently achieves strong and balanced performance despite being trained exclusively on MSRS. These cross-dataset results confirm that the semantic guidance mechanism generalizes effectively to unseen scene statistics, lighting conditions, and sensor configurations, demonstrating the cross-dataset robustness of the proposed approach.

### 4.5. Comparison of Model Complexity

**Figure 7:**
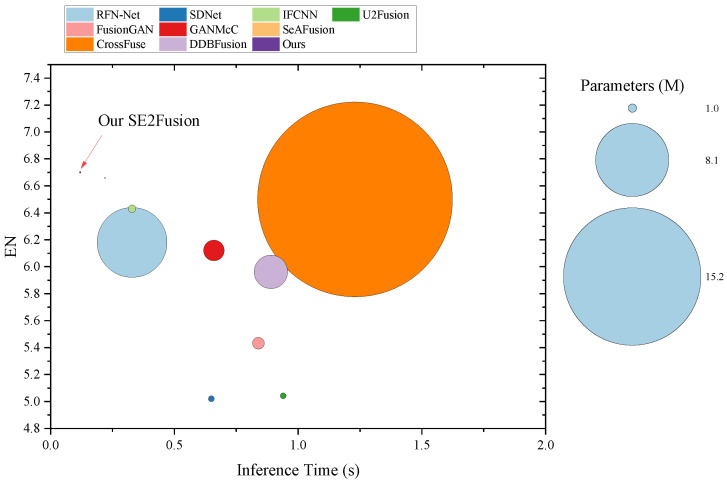
Comparison of model complexity with fusion performance.

We conduct a comprehensive efficiency comparison of different fusion methods, evaluating three key aspects: inference time, entropy (information preservation), and model parameters. As shown in Figure 7, the proposed method achieves the best overall efficiency–performance trade-off. Specifically, our method demonstrates the fastest inference speed among all compared methods. This efficiency advantage stems from the lightweight network architecture, which enables real-time processing capability, essential for practical applications. Additionally, the proposed method has the smallest model size, making it highly suitable for deployment on resource-constrained platforms. The compact architecture does not compromise fusion quality, demonstrating that our design achieves an excellent balance between model complexity and representation power.

### 4.6. Apllication in Object Detection

To further evaluate the effectiveness of the proposed fusion method in high-level vision tasks, we apply the fused images to an object detection pipeline. Specifically, we adopt the YOLOv5s detector. All fusion methods are evaluated under the same detection settings to ensure a fair comparison. The detection performance is measured using standard metrics, including Precision, Recall, mean Average Precision (mAP) at IoU thresholds from 0.5 to 0.95 (mAP@50-95), and F1-Score.

Table 4 summarizes the quantitative detection results. Our proposed method achieves the best overall performance across most metrics, with a Precision of 0.915, Recall of 0.852, mAP50-95 of 0.662, and F1-Score of 0.882. Compared to the raw infrared and visible images, our method improves the mAP@50-95 by 16.1% and 24.2%, respectively, demonstrating that the fused image provides more comprehensive information for accurate object detection.

### 4.7. Ablation Study

#### 4.7.1. The Analysis of Crucial Components

A controlled ablation study was conducted on the MSRS test set to isolate the contribution of each proposed component. Four reduced configurations are evaluated by systematically removing the SSIM loss term (*w/o SSIM*), the ESFA module (*w/o ESFA*), the ESFE module (*w/o ESFE*), and both semantic modules simultaneously (*w/o ESFA + ESFE*), with all other training settings kept identical. Results are presented in Table 5.

The full model achieves the best performance across all six metrics, confirming that every component makes a positive contribution to the overall fusion quality.

Removing the SSIM loss (*w/o SSIM*) causes a dramatic collapse in VIF from 0.89 to 0.46, the largest single-component degradation in the study, while other metrics remain close to full-model levels. This confirms that the SSIM constraint plays a decisive role in enforcing global structural consistency: without it, the network optimizes gradient and intensity objectives effectively but loses the ability to maintain overall structural coherence, severely degrading source-information fidelity.

Removing ESFA (*w/o ESFA*) produces declines across $Q^{AB/F}$, AG, and SF, with VIF also dropping markedly from 0.89 to 0.44. These degradations reflect the critical function of encoding-stage semantic priors: without ESFA, the network cannot distinguish semantically salient regions from background before cross-modal feature interaction, weakening both structural preservation and source-information fidelity in the fused output.

Removing ESFE (*w/o ESFE*) yields a similar pattern, with SF decreasing from 11.43 to 11.27 and VIF from 0.89 to 0.44. This demonstrates that decoder-stage semantic modulation is essential for restoring fine-grained textural detail during feature reconstruction.

When both modules are removed simultaneously (*w/o ESFA + ESFE*), the performance degradation is markedly more severe: $Q^{AB/F}$ falls from 0.86 to 0.67 and AG from 6.06 to 3.73, confirming a strong synergistic interaction between ESFA and ESFE. Without either module, the network reverts to a purely appearance-driven fusion strategy that lacks semantic discrimination capacity, losing the ability to balance target enhancement against background suppression at both encoding and decoding stages. Notably, unlike EN or SD, which measure global statistical diversity, VIF is grounded in a human visual system model and is particularly sensitive to contrast and edge preservation, which are precisely the properties enhanced by ESFA and ESFE. Hence, ablating these components causes a pronounced VIF drop while global statistical metrics remain nearly unchanged.

In summary, ESFA, ESFE, and the SSIM loss each provide distinct and complementary contributions: ESFA guides target-aware cross-modal feature interaction at the encoding stage; ESFE enables semantically conditioned detail recovery at the decoding stage; and the SSIM loss enforces structural perceptual consistency throughout training. Their combination is essential for the full model’s strong and balanced performance across all evaluation metrics.

#### 4.7.2. The Analysis of Feature Distribution

**Figure 8:**
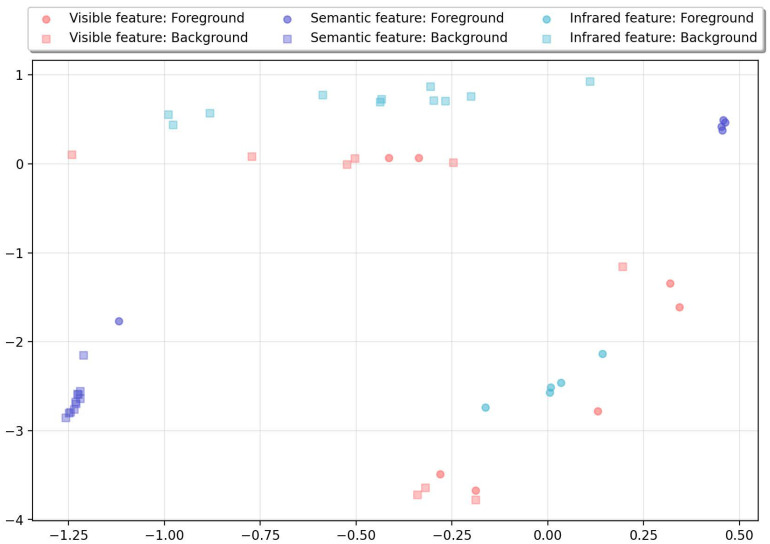
Comparison of t-SNE feature distributions between semantic features and raw IR/visible features.

To investigate the discriminative capability of the proposed semantic encoder, we conduct a feature distribution analysis by projecting the extracted features into a two-dimensional space using t-Distributed Stochastic Neighbor Embedding (t-SNE [40]). Specifically, we compare the feature distributions of three sources: semantic features from the proposed semantic encoder, visible features from the visible encoder, and IR features from the IR encoder. The foreground/background labels are obtained from the ground-truth masks from MSRS dataset, where pixels with value 0 are defined as background and all non-zero pixels as foreground. As illustrated in Figure 8, the semantic features exhibit a clear and well-separated distribution between foreground and background clusters. The foreground samples form a compact cluster with small intra-class variance, while the background samples are similarly concentrated in a distinct region. A large margin separates the two clusters, indicating that the semantic encoder effectively learns class-discriminative representations. This property is crucial for accurate target localization and complementary fusion, as it enables the model to easily distinguish objects from the surrounding environment.

#### 4.7.3. The Analysis of Hyper-Parameters

**Figure 9:**
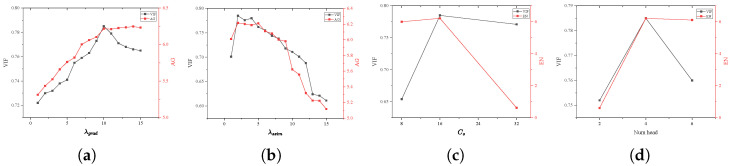
Quantitative comparison with different hyper-parameter settings. (**a**) $\lambda_{grad}$. (**b**) $\lambda_{ssim}$. (**c**) $C_{s}$. (**d**) Attention head numbers.

To investigate the influence of key hyper-parameters on the fusion performance, we conduct comprehensive ablation studies on the loss function weights, the semantic encoder channel number $C_{s}$, and the number of attention heads in the transformer module. As shown in Figure 9a,b, the gradient loss weight and SSIM loss weight are varied while keeping other settings fixed. The results indicate that setting the gradient weight to 10 and the SSIM weight to 2 yields the best performance, achieving a favorable trade-off between structural preservation and detail enhancement. Excessive weights on either term lead to over-smoothing or artifact introduction, confirming the necessity of proper balancing. On the other hand, the channel number of the semantic encoder determines the capacity of the feature representation. We evaluate three configurations: $C_{s}=8$, 16, and 32. As depicted in Figure 9c, the model with $C_{s}=16$ achieves the highest performance. When $C_{s}$ is too small (8), the encoder lacks sufficient representational power to capture complex semantic information. Conversely, when $C_{s}$ is too large (32), the model introduces redundant parameters without significant performance gains, potentially leading to over-fitting. Therefore, $C_{s}=16$ is selected as the optimal setting. Additionally, we also investigate the impact of multi-head attention configurations in the transformer module by varying the number of heads from two to six. As illustrated in Figure 9d, the model with four attention heads delivers the best fusion results. With too few heads (two), the attention mechanism fails to capture diverse feature relationships across channels. With too many heads (six), the model becomes over-parameterized and prone to optimization difficulties.

## 5. Conclusions

SPE2Fusion, a semantic prior-driven infrared and visible image fusion network, is presented to address the pervasive neglect of high-level semantic information in existing fusion methods. A lightweight semantic encoder implicitly extracts multi-scale scene priors without requiring segmentation supervision. These priors are injected at two complementary stages: the Efficient Semantic Feature Awareness module (ESFA) reinforces semantically salient regions during encoding, and the Efficient Semantic Feature Embedding module (ESFE) applies spatially adaptive normalization to modulate reconstruction during decoding. A cross-attention fusion block integrates complementary infrared and visible features under this dual semantic guidance, supervised by a multi-constraint loss combining gradient fidelity, intensity preservation, and structural similarity.

The core insight underlying SPE2Fusion’s effectiveness is that semantic priors serve as a unified guidance signal spanning the entire processing pipeline: from feature extraction (via ESFA) through image reconstruction (via ESFE). By injecting multi-scale semantic priors derived from a lightweight encoder, SPE2Fusion introduces an asymmetric weighting that reinforces semantically significant regions (pedestrians, vehicles, and other thermal targets) while suppressing background noise, which explains the consistent EN, AG, and SD gains observed across all three benchmarks.

A notable trade-off appears in the VIF metric, where SPE2Fusion (0.89) ranks third on the MSRS test set, behind SeAFusion (0.96) and GTF (0.93). VIF measures fidelity to the statistical properties of the source images as perceived by a human visual model. Semantic guidance intentionally deviates from strict source fidelity in order to enhance target saliency and structural coherence, and this trade-off is deliberate: the network prioritizes scene understanding over pure pixel preservation. Across the LLVIP and RoadScene benchmarks, SPE2Fusion’s VIF scores remain competitive, indicating that the semantic-fidelity tension does not worsen significantly under distribution shift.

The cross-dataset generalization results provide further evidence that the semantic prior mechanism captures a transferable property of infrared–visible scenes rather than overfitting to the MSRS distribution. The consistent superiority of SPE2Fusion across three substantially different scene distributions indicates that implicit semantic priors derived without segmentation supervision are robust to the illumination and context variations that characterize real-world deployment conditions.

The ablation study provides component-level evidence for the specific roles of ESFA and ESFE. Removing ESFA reduces SD from 43.44 to 42.64, indicating that encoding-stage semantic awareness is primarily responsible for contrast amplification in target regions. Removing ESFE reduces SF from 11.43 to 11.27, confirming that decoding-stage semantic modulation governs high-frequency texture recovery. Crucially, removing both modules simultaneously causes degradation far exceeding the sum of their individual effects ($Q^{AB/F}$ drops from 0.86 to 0.67; AG drops from 6.06 to 3.73), demonstrating genuine synergy between the two modules. Compared to recent transformer-based infrared–visible fusion approaches (such as the method of Zhao et al. [23], which addresses global–local feature dependency through cascaded dual interactive transformer modules and cross-attention fusion), SPE2Fusion pursues an orthogonal design axis by introducing top-down semantic priors as an explicit guide. The two directions are architecturally complementary, and their integration represents a natural avenue for extending the benefits of both paradigms.

Comprehensive experiments on the MSRS, LLVIP, and RoadScene benchmarks demonstrate that SPE2Fusion achieves leading performance across four of six quantitative metrics on MSRS, including EN (6.70), $Q^{AB/F}$ (0.86), AG (6.06), and SD (43.44), while maintaining competitive results on unseen datasets without domain adaptation. These findings substantiate a transferable fusion principle: incorporating high-level semantic priors as a unified guidance signal throughout the pipeline consistently improves information richness, edge fidelity, and contrast across diverse scene conditions.

Despite the promising performance achieved by our method, several limitations remain to be addressed. (1) Our current framework is primarily designed and validated for typical complex scenes (e.g., low light, overcast, and moderate environmental disturbances). Extreme conditions, including heavy noise, fog, snow, and rain, pose significant challenges and are left as one of our primary future directions. (2) To balance computational cost, our model is trained at 256×256 resolution. Although it generalizes reasonably to the 1K-resolution LLVIP test set, its performance and computational overhead on higher-resolution inputs (e.g., 2K and above) have not been systematically evaluated. We plan to investigate resolution-adaptive architectures and efficient inference strategies in future work. 

## Figures and Tables

**Table 1:** Quantitative comparison on the MSRS test set. Best results are shown in **red**, and second-best in **blue**.

Method	EN	$Q^{\mathrm{AB}/F}$	AG	SF	SD	VIF
RFN-Nest	6.185	0.387	2.121	6.167	29.076	0.652
SDNet	5.027	0.319	2.308	7.275	15.021	0.460
IFCNN	6.433	0.610	**3.911**	**12.019**	35.312	0.807
U2Fusion	5.042	0.333	2.251	7.206	20.212	0.480
FusionGAN	5.432	0.140	1.451	4.350	17.070	0.443
GANMcC	6.120	0.302	2.005	5.661	26.342	0.636
SeAFusion	**6.658**	**0.664**	3.711	11.122	**41.851**	**0.958**
CrossFuse	6.499	0.652	3.005	9.608	36.338	0.838
DDBFusion	5.962	0.521	2.567	7.813	25.895	0.588
**SPE2Fusion (Ours)**	**6.700**	**0.860**	**6.064**	**11.426**	**43.444**	0.894

**Table 2:** Quantitative comparison on 50 randomly selected images from the LLVIP dataset. Best results are shown in **red**, and second-best in **blue**.

Method	EN	$Q^{\mathrm{AB}/F}$	AG	SF	SD	VIF
RFN-Nest	7.003	0.275	2.360	6.814	37.905	0.614
SDNet	6.727	0.532	3.160	12.373	33.355	0.634
IFCNN	7.219	**0.648**	4.725	**16.234**	**53.586**	0.761
U2Fusion	6.451	0.435	3.217	10.423	31.163	0.580
FusionGAN	6.397	0.216	2.060	7.488	25.656	0.433
GANMcC	6.708	0.301	2.220	7.061	32.712	0.526
SeAFusion	**7.339**	0.634	**4.781**	**15.122**	50.588	**0.768**
CrossFuse	6.495	0.402	4.021	7.635	27.086	0.555
DDBFusion	6.807	0.772	4.936	9.128	34.111	0.691
**SPE2Fusion (Ours)**	**7.458**	**0.836**	**6.216**	14.336	**51.106**	**0.785**

**Table 3:** Quantitative comparison on 50 randomly selected images from the RoadScene dataset. Best results are shown in **red**; second-best in **blue**.

Method	EN	$Q^{\mathrm{AB}/F}$	AG	SF	SD	VIF
RFN-Nest	7.336	0.299	3.356	7.847	45.974	0.502
SDNet	7.058	0.507	5.132	12.806	37.672	0.571
IFCNN	7.122	**0.545**	5.477	**15.213**	39.185	**0.585**
U2Fusion	6.896	0.493	5.035	12.432	33.194	0.538
FusionGAN	7.068	0.259	3.383	8.732	39.096	0.367
GANMcC	7.430	0.199	2.651	5.694	**53.781**	0.411
SeAFusion	7.352	0.484	**7.702**	**20.210**	48.637	0.524
CrossFuse	7.190	0.724	4.407	11.996	45.042	0.569
DDBFusion	7.111	0.762	3.846	9.245	38.81	0.442
**SPE2Fusion (Ours)**	**7.439**	**0.769**	**5.480**	9.393	**53.989**	**0.621**

**Table 4:** The object detecion performance using different fusion images to YOLOv5s [39]. Best results are shown in **red**.

Images	Precision	Recall	mAP@50-95	F1-Score
Infrared	0.888	0.686	0.570	0.774
Visible	0.914	0.765	0.533	0.833
RFN-Net	0.906	0.796	0.632	0.847
SDNet	0.908	0.856	0.654	0.881
IFCNN	0.846	0.614	0.483	0.712
U2Fusion	0.907	0.801	0.611	0.851
FusionGAN	0.911	0.824	0.643	0.865
GANMcC	0.901	0.853	0.645	0.876
SeAFusion	0.908	0.847	0.656	0.876
CrossFuse	0.912	0.820	0.605	0.864
DDBFusion	0.908	0.848	0.652	0.877
**SPE2FUsion (Ours)**	**0.915**	**0.852**	**0.662**	**0.882**

**Table 5:** Ablation study of SPE2Fusion on the MSRS test set. Best results are shown in **red**.

Configuration	EN	$Q^{\mathrm{AB}/F}$	AG	SF	SD	VIF
*w/o SSIM*	6.681	0.851	6.014	11.315	43.387	0.459
*w/o ESFA*	6.683	0.850	6.021	11.391	42.639	0.443
*w/o ESFE*	6.672	0.852	6.062	11.267	42.186	0.438
*w/o ESFA + ESFE*	6.683	0.673	3.725	11.244	42.725	0.393
**Full model (ALL)**	**6.700**	**0.860**	**6.064**	**11.426**	**43.444**	**0.894**

## Data Availability

Data are contained within the article.
